# Asthma and atopy in children born by caesarean section: effect modification by family history of allergies – a population based cross-sectional study

**DOI:** 10.1186/1471-2431-12-179

**Published:** 2012-11-16

**Authors:** Ourania Kolokotroni, Nicos Middleton, Marina Gavatha, Demetris Lamnisos, Kostas N Priftis, Panayiotis K Yiallouros

**Affiliations:** 1 Cyprus International Institute for Environmental and Public Health in Association with Harvard School of Public Health, Cyprus University of Technology, Limassol, Cyprus; 2 School of Nursing, Cyprus University of Technology, Limassol, Cyprus; 3 St George University of London Medical School at the University of Nicosia, Nicosia, Cyprus; 4 Archbishop Makarios III Hospital, Nicosia, Cyprus; 5 Third Department of Pediatrics, University of Athens School of Medicine, Attikon Hospital, Athens, Greece

**Keywords:** Asthma, Vaginal delivery, Caesarean section, Wheeze, Atopic sensitization, Child

## Abstract

**Background:**

Studies on the association of birth by caesarean section (C/S) and allergies have produced conflicting findings. Furthermore, evidence on whether this association may differ in those at risk of atopy is limited. This study aims to investigate the association of mode of delivery with asthma and atopic sensitization and the extent to which any effect is modified by family history of allergies.

**Methods:**

Asthma outcomes were assessed cross-sectionally in 2216 children at age 8 on the basis of parents’ responses to the ISAAC questionnaire whilst skin prick tests to eleven aeroallergens were also performed in a subgroup of 746 children. Adjusted odds ratios of asthma and atopy by mode of delivery were estimated in multivariable logistic models while evidence of effect modification was examined by introducing interaction terms in the models.

**Results:**

After adjusting for potential confounders, children born by C/S appeared significantly more likely than those born vaginally to report ever wheezing (OR 1.36, 95% CI 1.07-1.71), asthma diagnosis (OR 1.41, 95% CI 1.09-1.83) and be atopic (OR 1.67, 95% CI 1.08-2.60). There was modest evidence that family history of allergies may modify the effect of C/S delivery on atopy (p for effect modification=0.06) but this was not the case for the asthma outcomes. Specifically, while more than a two-fold increase in the odds of being a topic was observed in children with a family history of allergies if born by C/S (OR 2.62, 95% CI 1.38-5.00), no association was observed in children without a family history of allergies (OR 1.16, 95% CI 0.64-2.11).

**Conclusions:**

Birth by C/S is associated with asthma and atopic sensitization in childhood. The association of C/S and atopy appears more pronounced in children with family history of allergies.

## Background

In recent decades, there has been an increase in the proportion of children born by caesarean section (C/S) beyond the recommended by WHO level of 15%. Rises were mostly observed in developed nations such as the United States of America [1], United Kingdom [2] and other European countries [3]. In England, for example, in year 2010, 23.4% of singletons were delivered by C/S [4]. In Cyprus, the Perinatal Health Survey performed in year 2007 by the Statistical Services of the country estimated the C/S rate to be as high as 50.9% [5]. During this period, a rise in the prevalence of asthma and allergies was also observed and, to date, a number of studies have examined a possible link between allergic diseases and mode of delivery [6-13]. The potential effect of C/S on asthma and allergies is thought to be mediated through immunological mechanisms, mainly focusing on the fact that children born by C/S have reduced exposure to vaginal microbial flora. Differences in the exposure to maternal vaginal or intestinal flora have been shown to be associated with alteration in the neonatal gut microflora [14,15] and neonatal cytokine response patterns [16] which can subsequently lead to changes in the Th1/Th2 helper cells balance and the risk of developing atopy [17-19]. However, epidemiological studies investigating whether the risk of developing allergic conditions is associated with mode of delivery have not always produced consistent findings [6,8].

Two meta-analyses, both published in 2008, summarized the existing evidence. Reviewing a total of 20 articles, the study by Thanagnanam et al. reported a statistically significant 20% increase in the risk of developing asthma in children born by C/S [20]. The second meta-analysis by Bager et al. [21] summarized across a total of 26 studies the association of C/S with 6 different allergic outcomes including food and inhalant atopy. It concluded that only 1-4% of the increase in the allergic outcomes studied can be attributed to C/S delivery whilst no association was observed between C/S delivery and inhalant atopy and atopic dermatitis. Interestingly, both groups of authors noted the lack of studies that examined the association in non-atopic and atopic asthmatics separately. It is possible that this association may be different in the atopic and non-atopic asthma phenotypes since research evidence suggests that risk factors associated with asthma might be different for each asthma phenotype. Indeed, the results of a more recent longitudinal study [22], showed that C/S delivery was positively associated with allergic rhinitis and atopy, but not asthma, among 432 children aged 9 years with a parental history of atopic diseases,. Interestingly, another prospective study from the Netherlands followed 2917 children from birth to 8 years of age and showed that the association of C/S delivery with asthma was stronger in children of two atopic parents than in children of non-allergic parents whilst in contrast to the previous study, no association was shown with atopy [23]. Therefore the evidence on whether the association between C/S delivery and allergic diseases is dependent on atopic background is so far limited and contrasting. In this study, we aimed to investigate the association between asthma outcomes and atopic sensitization with mode of delivery amongst 7–8 year old children and assess whether any effect of C/S delivery is differential in children with and without a family history of allergies.

## Methods

### Study population and design

In year 2008, we invited all children attending the second grade of public primary schools (i.e. 7–8 years of age, n=4583) in the two most populous districts of the Republic of Cyprus (i.e. Nicosia and Limassol) to participate in a cross sectional study which primarily aimed to reassess asthma and allergy prevalence in Cyprus. Parents of participating children were asked to complete the study questionnaire which consisted of the Greek version of the ISAAC core questionnaire and a series of questions on demographic and lifestyle characteristics. The following year, we invited a randomly selected sample amongst the participating children of the original survey who resided in the district of Nicosia to attend Makarios Children’s Hospital (the tertiary paediatric hospital in Cyprus) in order to undergo amongst other assessments skin prick testing (SPT) to 11 aeroallergens (as described below). The study was approved by the Cyprus National Bioethics Committee. Written consent was obtained from the parents or legal guardians of all study participants.

### Definition of mode of delivery

A question on the child’s mode of delivery was included in the study questionnaire by which the parents provided information on whether the child was born either by vaginal delivery or by C/S. For the purposes of the statistical analysis, mode of delivery was considered a binary variable as no distinction could be made from the available information between emergency or elective C/S.

### Definition of asthma outcomes

Based on answers given to the relevant questions in the ISAAC core questionnaire, we studied the following self-reported asthma outcomes: (a) Ever Wheeze (Has your child ever had wheezing or whistling in the chest at any point in time?), (b) Current Wheeze (Has your child had wheezing or whistling in the chest in the past 12 months?), (c) Ever Asthma (Has your child ever had asthma?) as well as (d) the composite measure Active Asthma, which was defined as a positive response to having Ever Asthma together with a positive response regarding Current Wheeze.

### Definition of atopic sensitization

We performed SPT on the volar aspect of the lower arm of the children whose parents consented to the specific test. Children who had (a) personal history of an anaphylactic reaction in the past and/or (b) wheezing on auscultation on the day of the examination and/or (c) an acute asthma attack in the preceding four weeks and/or (d) a personal history of severe cardiac conditions were excluded from SPT for safety reasons.

For SPT we used extracts for eleven aero-allergens (Greer Allergy immunotherapy) that are considered common in Cyprus: house dust mite (Dermatophagoides farinae and Dermatophagoides pteronyssinus), olive (olea europaea), mugwort (artemisia vulgaris), weed mix (kochia scoparia/ chenopodium album/ salsola kali), grass mix (poa pratensis/ dactylis glomerata/ phleum pretense), bahia grass (paspalum notatum), mold mix (alternaria tenuis/ aspergilus niger/ helminthosporidium sativum/ hormodendrum hordei/ penicillum chrysogenum), cat hair (felis catus), dog epithelia (canis familiaris), mouse epithelia (mus musculus) and cockroach mix (periplaneta americana/ blatella germanica). The procedure was standardized using histamine as a positive and glycerinated saline as a negative control. We considered a SPT result as positive if the wheal diameter was 3mm or larger after we subtracted the diameter of the negative control wheal. A child was considered as having atopic sensitization if he or she had a positive SPT to at least one of the above allergens.

### Definition of other variables

In order to control for the potential confounding effect of socio-demographic and/or other risk factors on the association between mode of delivery and asthma/atopy outcomes, the following a priori selected variables were considered in multivariable analyses: sex, country of birth (Cyprus or elsewhere), district of residence (i.e. Nicosia or Limassol), urban/rural environment, sibling order (first-, second, third or higher born), parental level of education (defined as the highest level of educational attainment by at least one of the parents: primary, secondary or tertiary education), exclusive breastfeeding for at least 2 months, nursery attendance in the first year of life, sharing bedroom with older siblings any time from birth up to age 5, current exposure to parental smoking at home, history of maternal smoking during pregnancy, low birth weight (less than 2500 gram) and family history of allergy (i.e. whether father and/or mother and/or sibling reportedly had a history of asthma and/or eczema and/or hayfever).

### Statistical analysis

We calculated prevalence estimates (overall as well as stratified by mode of delivery) for each of the asthma outcomes by dividing the number of positive responses to the given question in the ISAAC questionnaire by the total number of participants expressed as percentages; missing or inconsistent values were taken to be a negative response. Prevalence estimates of atopic sensitization were calculated by dividing the number of children identified as having a positive SPT to at least one allergen by the total number of children that underwent the test. The 95% confidence intervals around all the prevalence estimates were constructed using the normal large-sample approximation for the standard error of a single proportion. The representativeness of the sub-group of participants (i.e. children who responded to the call to participate in the second round of hospital assessments including the SPT) in terms of their socio-demographic characteristics was assessed in chi-squared tests against the total sample.

Odds ratios (OR’s) of asthma and atopy outcomes under study by mode of delivery were estimated in multivariable logistic models adjusting for the effect of potential confounders known a priori to be associated with outcomes and exposures. As information regarding the gestational age at birth was not available, the birth weight of the participants was included in the models. In addition, all analyses were repeated after excluding children with a birth weight of less than 2.5 Kg. Finally, we assessed for any evidence of modification in the magnitude of effect in children with and without family history of allergy by including an interaction term between family history of allergy and mode of delivery. Evidence for effect modification was assessed in Likelihood Ratio Tests comparing the goodness of fit of models with and without the interaction term. All statistical analyses were performed using the SPSS 18 software package. Statistical significance was set at p < 0.05.

## Results

The characteristics of the participating children are presented in Table 1. Out of the 4583children invited, we obtained completed questionnaires from 2216 children aged 7–8 years (representing 48% of all children in this age range attending public primary schools in the districts of Nicosia and Limassol). Reasons for low participation include the increasing reluctance of parents to offer personal information and the rise in the number of surveys among the school population in Cyprus. There were about equal numbers of boys and girls while the vast majority of participants were born in Cyprus. As many as 31.7% of the participants were born by C/S, a percentage lower than the one reported by the only Perinatal Health Survey performed on the island in the year 2007, which might reflect a worrying increase in the percentage of children born by CS in Cyprus in less than a decade. More than 1 in 3 children (37%), as reported by their parents, had a positive family history of allergies while 39% were currently exposed to tobacco smoke at home.

**Table 1:** Socio-demographic characteristics of participating children

		**All Participants (Nicosia & Limassol)**	**Sub group of children participating in SPT**
		**N=2216**	**N= 797**
**Participant Characteristics (%)**			
Sex	Male	50.5	50.3
Country of Birth	Cyprus	95.3	95.4
Sibling Order	1^st^	38.7	39.4
	2nd	34.3	33.7
	3^rd^ or more	27.0	26.9
Family history of Allergies	Yes	37.0	41.8**
Urban/rural residence	Urban	66.5	64.7
Highest level of parental education	None or Primary	1.4	1.0
	Secondary	44.9	40.2
	Tertiary	53.7	58.8**
Parental smoking	Yes	39.0	36.7
Mother smoking during pregnancy	Yes	2.9	2.8
Mode of delivery	Normal	68.3	68.1
	Caesarean	31.7	31.9
Birth weight <2500gr	Yes	13.9	14.3
Exclusive Breastfeeding for > 2 months	Yes	40.9	46.1**
Nursery attendance in first year of life	Yes	9.2	9.6
Bedroom sharing	Yes	37.4	38.5

*, ** P-value <0.05 and <0.01 respectively in chi-squared test comparing the sub-group who underwent SPT with all participants.

A total number of 797 children from Nicosia took part in hospital assessments including performance of SPT. This corresponds to 71% of the children that resided in Nicosia and took part in the ISAAC survey. We assessed allergic sensitization in 746 of the 797 children. Fifty one children were excluded from undergoing this test either due to parental refusal to consent for the specific test and/or because they met one or more of the exclusion criteria.

No significant differences in terms of most of the socio-demographic and risk characteristics were observed between the subgroup of children that participated in the hospital assessments and the rest of the children who only participated in the questionnaire part of the study. Nevertheless, a significantly higher proportion of children who participated in hospital assessments were breastfed for more than two months (46.1% vs 40.9%, p<0.01), reported a positive family history of allergies (41.8% vs 37%, p<0.05) and had at least one parent who attained tertiary education level (58.8% vs 53.7%, p<0.05).

In Table 2, we present the prevalence estimates of asthma/atopy outcomes by mode of delivery as well as the association between mode of delivery and the outcomes under study expressed as OR’s as estimated in univariable and multivariable logistic models. In the unadjusted models, the odds of all asthma outcomes appear significantly increased in children born by C/S compared to those born vaginally. After adjusting for all potential confounders in multivariable models, the observed estimates attenuate slightly in all cases but associations remain statistically significant for two out of the four outcomes investigated here i.e. ever wheeze (OR: 1.36, 95% CI: 1.07-1.71) and ever asthma (OR: 1.41, 95% CI: 1.09-1.83). In the subgroup of children that underwent SPT, the prevalence of atopic sensitization was higher in children born by C/S compared to those born vaginally (18.5% vs 13.7%, p-value<0.05). After adjusting for potential confounders, there was evidence of significantly higher odds of atopic sensitization at the age 8–9 years in those born by C/S (OR: 1.67, 95% CI: 1.08-2.60). When the analyses were repeated after the exclusion of children with a birth weight lower than 2.5 Kg (results not shown in detail), inferences remained unaffected or attenuated slightly whilst the confidence intervals around the effect estimates became as expected slightly wider due to the reduced sample size. For instance, the odds ratio of ever asthma in children born by C/S compared to vaginally in this group was 1.39, (95% CI: 1.05-1.85) whilst for ever wheeze and atopic sensitization were 1.40 (95% CI: 1.08-1.80) and 1.59 (95% CI: 0.99-2.57) respectively.

**Table 2:** Association between mode of delivery and asthma and allergy outcomes at age 8–9 years

**Outcomes**	**Total Population**	**Prevalence (95% CI)**		**Odds ratios by mode of delivery (Born by C/S Vs vaginally)**	
		**Born vaginally**	**Born by C/S**	**Unadjusted OR (95% CI), p-value**	**Adjusted OR† (95% CI), p-value**
Whole sample*	n=2216	n= 1500	n= 695		
Ever Wheeze	25.0	23.1	29.5	1.38 (1.13-1.69),	1.36 (1.07- 1.71)
	(23.2-26.8)	(21.0-25.2)	(26.1-32.9)		
Current Wheeze	8.6	7.5	11.1	1.53 (1.13-2.08)	1.23 (0.87- 1.74)
	(7.4-9.8)	(6.2-8.8)	(8.8-13.4)		
Ever Asthma	17.6	15.9	21.3	1.44 (1.14-1.80)	1.41 (1.09- 1.83)
	(16.0-19.2)	(14.0-17.8)	(18.3-24.3)		
Active Asthma	5.7	5.1	7.2	1.44 (1.00-2.08)	1.23 (0.81- 1.88)
	(4.7-6.7)	(4.0-6.2)	(5.3-9.1)		
Sub-group*	n=746	n= 507	n= 354		
Atopic Sensitization	15.1	13.7	18.5	1.47 (0.98- 2.20)	1.67 (1.08- 2.60)
	(12.6-17.6)	(10.8-16.6)	(13.7-23.3)		

† Adjusted for sex, country of birth, district, urban/rural residence, birth order, parental level of education, parental smoking at home, mother smoking during pregnancy, exclusive breastfeeding for more than 2 months, birth weight <2500 gr., nursery attendance in the first year of life, bedroom sharing with older sibling, and family history of allergy. * Numbers reflect missing values on some variables.

Table 3 presents the effect estimates of the association of mode of delivery with asthma and atopic sensitization stratified by family history of atopy. Effect estimates for asthma outcomes were generally of similar magnitude in those with and without family history of atopy both before and after adjustments. In contrast, there was modest evidence of effect modification by family history of atopy in the case of atopic sensitization (p value for Likelihood Ratio Test for effect modification=0.06). Even though statistical significance was slightly short of significance at the 5% level, pronounced differences in the magnitude of effect between the two groups were observed. In children with a family history of atopy, the odds of atopic sensitization was increased by at least two-fold in those born by C/S compared to those born vaginally (OR 2.62, CI: 1.38-5.00). In contrast, this does not seem to be the case in children with no family history of atopy where no association with mode of delivery was observed (OR 1.16, CI: 0.64-2.11).

**Table 3:** Prevalence and odds ratios (and 95% CI) of asthma and atopic sensitization at age 8–9 by mode of delivery, stratified by family history of allergy

	**NO FAMILY HISTORY OF ALLERGY**				**FAMILY HISTORY OF ALLERGY**				**Effect modification p-value‡**
	**Vaginal Prevalence %(95%CI)**	**Caesarean Prevalence % (95%CI)**	**Unadjusted Odds Ratios (95% CI)**	**Adjusted Odds Ratios† (95% CI)**	**Vaginal Prevalence % (95%CI)**	**Caesarean Prevalence % (95%CI)**	**Unadjusted Odds Ratios (95% CI)**	**Adjusted Odds Ratios† (95% CI)**	
All children*	**N=929**	**N=413**			**N=531**	**N=259**			
Ever Wheeze	15.2(12.9-17.5)	19.9 (16.0-23.8)	1.38 (1.02-1.87)	1.45 (1.05-2.00)	37.3 (33.2-41.4)	44.8 (38.7-50.9)	1.36 (1.00-1.83)	1.27 (0.92-1.75)	0.55
Current Wheeze	4.1 (2.8-5.4)	6.8 (4.4-9.2)	1.70 (1.03-2.82)	1.54 (0.89-2.64)	13.7 (10.8-16.6)	17.8 (13.1-22.5)	1.37 (0.92-2.06)	1.07 (0.69-1.66)	0.31
Ever Asthma	10.5 (8.5-12.5)	13.3 (10.0-16.6)	1.30 (0.91-1.84)	1.40 (0.96-2.05)	25.6 (21.9-29.3)	34.4 (28.6-40.2)	1.54 (1.11-2.12)	1.42 (1.00-2.01)	0.96
Active Asthma	2.7 (1.7-3.7)	3.4 (1.7-5.1)	1.26 (0.65-2.46)	1.19 (0.57-2.46)	9.6 (7.1-12.1)	13.5 (9.3-17.7)	1.49 (0.94-2.36)	1.25 (0.76-2.07)	0.90
Sub-group*	**N=289**	**N=137**			**N=204**	**N=89**			
Atopic sensitization	14.8 (10.8-18.8)	15.0 (9.2-20.8)	1.04 (0.60-1.81)	1.16 (0.64-2.11)	12.1 (7.8-16.4)	24.2 (15.8-32.6)	2.42 (1.30-4.96)	2.62 (1.38-5.00)	0.06

† Adjusted for sex, country of birth, district of residence, urban/rural residence, birth order, parental level of education, parental smoking at home, mother smoking during pregnancy, birth weight < 2500 gr., exclusive breastfeeding for more than 2 months, nursery attendance in the first year of life, bedroom sharing with older sibling. ‡ P-value for effect modification by family history of allergy in multivariable models. * Numbers reflect missing values on some variables.

## Discussion

### Main findings

We investigated whether delivery by C/S is associated with asthma and atopy outcomes at age 7–9 years and assessed whether this relationship differs in children with and without a family history of allergic diseases. After adjusting for potential confounders, positive associations of C/S delivery with ever wheeze, ever asthma diagnosis and atopic sensitization were observed. While no effect modification by family history of atopy was observed in terms of the asthma outcomes, there was some evidence that the association of C/S with atopic sensitization was particularly pronounced in the presence of family history of allergies. In fact, among those without family history of allergies, atopic sensitization was not found to be associated with C/S.

### Limitations

Despite the cross sectional design of the study, the temporal sequence of the exposure and outcomes in this study is clear. In addition the self – reporting nature of the mode of delivery is unlikely to have lead to misclassification of the exposure as parents usually report accurately the mode of delivery of their child. Of course, information bias in terms of self reporting asthma outcomes as well as other covariates (e.g. family history of allergies or birth weight) cannot be excluded. Nevertheless, there is no reason to believe that any such bias should occurred differentially with regards to the mode of delivery. Even though the participation rate in the questionnaire survey was rather low, the final sample corresponds to about half of all children in this age range. While it is not unlikely that asthmatic children may have been more inclined to participate in the study and may have been overrepresented in the sample (and as a result inflate the prevalence estimates), there is no reason to believe that the participation rate could have affected the study outcomes differentially in terms of mode of delivery (i.e. there is no reason why more asthmatics born by C/S or more non-asthmatics born vaginally would have participated in the study). Another limitation of our study is the lack of available information on some potential confounding factors such as transient tachypnea of the newborn [24], pregnancy complications (e.g. prematurity and intrauterine growth retardation -IUGR) and delivery complications. However, in view of the fact that many of these variables as well as birth weight are strongly correlated [25], it is not uncommon for studies to avoid adjusting for all, especially if adding them to the model does not seem to affect the findings [23]. In this study, we used low birth weight as a surrogate parameter primarily for both prematurity and IUGR and all analyses were repeated after exclusion of children with a low birth weight. Nevertheless, we adjusted in our model for other important confounding factors such as maternal smoking during pregnancy and exclusive breastfeeding for longer than two months and parental educational level [26]. Finally it should be noted that we only investigated atopic sensitization using inhalant allergens and have not included food allergens in our assessment although summary of evidence so far has shown the association of C/S delivery with atopic sensitization to be stronger in the case of food allergens than inhalant allergens [21] and hence this association would have probably been stronger should we have included food allergens in our panel of antigens in SPT’s.

### Association between delivery by C/S and asthma

A number of epidemiological studies have investigated the risk of having asthma in individuals born by C/S. Two recent meta-analyses have reported a modest increase in the risk of asthma in children born by C/S [20,26]. In this study, we have also shown associations between delivery by C/S and all asthma outcomes investigated. Specifically, in the case of ever wheeze and ever asthma, the effect estimates did not attenuate after adjusting for confounders and the observed associations remained statistically significant.

Due to unavailability of data, it was not possible to investigate the association of CS with asthma outcomes differentially in children born by elective and emergency CS. Likewise, it would have been preferable to adjust the observed association for prematurity; nevertheless in our study inferences remain unaffected both when adjusting for birth weight (as a surrogate for prematurity) as well as when the analyses are restricted to those with a birth weight higher than 2.5 Kg. Furthermore, prematurity in the literature has been associated with increased risk of asthmatic symptoms but not with the development of allergies [27] and thus is unlikely to confound the observed association of C/S with atopic sensitization.

In both meta analyses, the authors commented on the inconsistency of findings in the literature and suggested that to some extent the observed heterogeneity may be attributed to differences in methodology, study designs and the populations under study (adults vs children), use of different asthma outcome definitions, the effect of bias and lack of appropriate adjustment for confounders, limited statistical power due to small sample size etc. It is also likely that the association of delivery by C/S and asthma differs in atopic and non atopic children and, therefore, by not distinguishing on the basis of atopic background effect estimates may be reflecting a mixed picture.

Indeed, the study by Roduit et al. [23] found that the association of C/S and asthma was more pronounced in children with one or two allergic parents, although no test for effect modification was provided. In contrast, our analysis did not reveal any difference in the estimates of the effect of C/S delivery on any of the asthma outcomes in children with and without family history of allergies indicating probably that the positive association of CS delivery with asthma outcomes found in our study is important in both children with and without family history of allergies. On the contrary, the findings of the longitudinal study by Pistiner et al. did not find any evidence of a positive association of C/S delivery and asthma disease or symptom in 432 nine year old children with parental history of atopy. Therefore, the effect of genetic predisposition on the association of asthma with C/S delivery remains unclear and further research is needed to address this potential interaction.

### Association between delivery by C/S and atopic sensitization

Nine studies to date, all prospective in nature, have investigated the potential association of atopic sensitization with C/S delivery using objective methods i.e. food challenge [28], IgE specific levels [9,23,29] and SPT [1,3,15,16,30] to food and/or inhalant allergens. While some of these studies have a small sample size [6] or have tested for only a small number of allergens [31], the majority could not replicate an association between delivery by C/S and atopic sensitization [6,8,9,23,31,32]. A recent review [33] as well as one of the meta-analyses [21] could not verify this association and concluded that, if anything, delivery by C/S might be associated with food but not with inhalant atopy.

Four of these studies examined the potentially modifying effect of family history of atopy or, alternatively, restricted their investigations to children born to atopic parents [8,21,23,28] with mixed findings. Maitra et al. found that the relationship between C/S delivery and atopy was not differentially affected by maternal history of hayfever amongst 5,916 seven year-old children in England [8]. However, a degree of misclassification cannot be excluded since only maternal history of hayfever was considered and neither asthma nor eczema were included in the definition used. Roduit et al. showed no significant association of C/S with atopy [23]. Even though, as mentioned earlier, the authors reported that the association between C/S and asthma was more pronounced amongst children of allergic parents, they found the opposite to be true for atopy with a more pronounced effect in children of non-allergic parents. Nevertheless, it is not known to what extent the particularly low rates of C/S in this population together with the differential non-participation rate observed in this study (with higher loss to follow up amongst the subjects of the atopic background group) may have possibly affected the direction of estimates.

In contrast, Pistiner et al. showed that C/S was associated with a two-fold increase in the odds of atopy (OR 2.1, CI 1.1-3.9, p=0.02) in children with parental history of atopy [22]. In addition, the study by Eggesbo et al. showed that the risk of having a positive food challenge to an egg was 7 times higher in those born by C/S to allergic mothers compared to those born vaginally to non allergic mothers amongst 2803 two year-old Norwegian children [23]. In agreement with these findings in this study, we also found evidence of an overall association between C/S delivery and atopy. In addition, we showed a modest evidence to suggest that this association might not be independent of family history of allergies as children with a family history of atopy have 2.6 times the odds of having atopic sensitization at age 8–9 years if born by C/S while no similar association was observed in children with no family history of allergies.

The differential effect of atopic background has also been studied in relation to other early life environmental exposures and asthma development [34]. Indeed, maternal overweight has been shown to be associated with an increased risk of asthma in children predisposed to asthma at the age of 8 years whilst no association was observed in children without predisposition. The differential effect of atopic background might be suggestive of gene-environment interaction that can influence the development of the immune system. It has been previously shown that atopic and non-atopic children develop different T-helper lymphocyte responses during the first year of life [35]. More recently, a study investigating the interaction of day care exposure in the first year of life with 72 genetic polymorphisms at 45 candidate loci demonstrated 22 significant interactions of 16 polymorphisms with day care attendance on cytokine profiles and atopic phenotypes [36]. Furthermore, there is growing evidence that gene-environment interactions are also involved in the development of childhood food allergy [37]. Therefore, the interaction of genetic predisposition with early life environmental exposures, such as in the case of delivery by C/S, might play an important role in the development of allergic and probably other immune related conditions; this will need to be explored further in future studies.

## Conclusion

Birth by C/S is associated with asthma and atopic sensitization in Cypriot children aged 8–9 years. The association of C/S delivery with atopic sensitization seems to be more pronounced in children with family history of allergy but this is not the case for asthma. Further studies are needed to investigate the potential interaction of genetic predisposition with C/S delivery and their effect on immune responses and asthma/atopy development in childhood.

## Competing interests

The authors declare that they have no competing interest.

## Authors’ contributions

OK supervised acquisition of the data, performed the statistical analysis and prepared the first draft of the manuscript. PY conceived, designed and supervised the conduction of the study. NM and DL advised with the statistical analysis. PY and NM assisted in drafting the manuscript. MG helped in data collection and interpretation. KNP critically revised the manuscript. All authors have read and approved the final version of the manuscript.

## Pre-publication history

The pre-publication history for this paper can be accessed here:

http://www.biomedcentral.com/1471-2431/12/179/prepub
